# Complete response in unresectable HER2+ cholangiocarcinoma with histotripsy and systemic therapy followed by liver transplantation: a case report

**DOI:** 10.3389/fonc.2026.1740407

**Published:** 2026-10-07

**Authors:** Tiffany M. Juarez, Jimmy Ton, Ivan Babin, Chase J. Wehrle, Victoria Chuchua, Santosh Kesari, Bin S. Teh, Sadhna Dhingra, Ashish Saharia, Ashton Connor, Sudha Kodali, Kevin Burns

**Affiliations:** 1 Leonard Cancer Institute, Providence Mission Hospital, Mission Viejo, CA, United States; 2 Department of Interventional Radiology, Providence Mission Hospital, Mission Viejo, CA, United States; 3 Liver Transplantation & HPB Surgery, Cleveland Clinic Foundation, Cleveland, OH, United States; 4 Department of Neuro-Oncology, Providence Mission Hospital, Mission Viejo, CA, United States; 5 Department of Radiation Oncology, Houston Methodist Hospital, Houston, TX, United States; 6 Department of Pathology and Genomic Medicine, Houston Methodist Hospital, Houston, TX, United States; 7 JC Walter Jr Center for Transplantation, Houston Methodist Hospital, Houston, TX, United States; 8 Sherrie and Alan Conover Center for Liver Disease and Transplantation, Houston Methodist Hospital, Houston, TX, United States

**Keywords:** bile duct cancer, cholangiocarcinoma, Edison System, histotripsy, liver cancer, liver transplant

## Abstract

Intrahepatic cholangiocarcinoma carries a poor prognosis making its management challenging. Histotripsy is a non-invasive, non-ionizing, and non-thermal therapy that mechanically disrupts tumors through acoustic cavitation. We present the case of a 59-year-old man with locally advanced, multifocal, stage II intrahepatic cholangiocarcinoma who achieved a complete pathologic response in the explanted liver after treatment with histotripsy and systemic drug therapy consisting of gemcitabine, cisplatin, durvalumab, and trastuzumab deruxtecan. This case highlights the potential role of noninvasive histotripsy as a bridging therapy to transplantation in selected patients with cholangiocarcinoma.

## Introduction

Intrahepatic cholangiocarcinoma is a rare, aggressive cancer of the intrahepatic biliary tree, marked by a poor prognosis due to an insidious clinical presentation that frequently leads to late diagnosis and limited treatment options (1). The location of the tumor(s) often complicates surgical removal, and symptoms typically appear at advanced stages of the disease, making early detection and effective treatment challenging. Standard treatments include surgery, which offers the best chance of a cure if the cancer is diagnosed early, chemotherapy, radiation, and more recently, immunotherapy (2, 3). Palliative care, including biliary drainage and stenting, remains crucial to provide symptomatic relief and improve quality of life. The aggressive nature and high mortality rate of cholangiocarcinoma make it an extremely difficult disease to manage, requiring a multidisciplinary team approach. Although many patients with cholangiocarcinoma ultimately develop extrahepatic disease, most die of intrahepatic progression or from complications related to recurrent biliary obstruction such as infection in the form of cholangitis or an inability to receive further therapy due to elevated liver enzymes, especially bilirubin. Therefore, there is an urgent need to develop more effective treatment strategies to control or treat hepatic progression and prevent or relieve biliary obstruction.

Histotripsy is a novel, non-ionizing, non-invasive, and non-thermal image-guided therapy that uses very short (micro-second duration) ultrasound pulses to generate acoustic cavitation from endogenous gas in tissue resulting in rapid expansion and collapse of microbubbles (bubble cloud) that can be precisely applied to mechanically destroy tumors. The bubble cloud mechanically liquifies the targeted tissue at a focal point into an acellular homogenate that is generally resorbed within a few months but loses its effect on tissue below a certain threshold thereby enhancing the safety profile of histotripsy. Because the bubble cloud creates a purely mechanical process that destroys tissue within the spatial location and volume of the bubble cloud only, non-targeted/adjacent tissues remain unaffected (4, 5). For patients with cholangiocarcinoma, histotripsy offers several potential benefits. Its non-invasive nature reduces recovery times associated with traditional surgical or other image-guided interventions. Histotripsy is delivered in one fraction, and patients often go home the same or next day, allowing them to continue receiving systemic therapy (6). Furthermore, histotripsy can be employed in cases where surgical resection is not feasible due to the tumor’s location or the patient’s health status. An additional technical advantage of histotripsy is its ability to precisely target tumors adjacent to collagen-rich structures, such as bile ducts and blood vessels, thereby preserving treatment efficacy in challenging anatomical regions. Clinical studies to date have demonstrated an excellent safety profile and promising technical efficacy (7–10).

Here, we report a patient with locally advanced intrahepatic cholangiocarcinoma who responded extremely well to a combination approach consisting of targeted systemic agents (including gemcitabine, cisplatin, durvalumab, and trastuzumab deruxtecan) with locoregional histotripsy as a bridge to liver transplant. Pathology of explanted liver showed four tumors, all of which showed pathologic complete response highlighting the potential benefit of this combined approach.

## Case presentation

A 59-year-old male with a history significant for hypotension, hypothyroidism, and coronary artery disease presented with right upper quadrant abdominal pain and recurrent fever, without clinical signs of biliary obstruction such as scleral icterus or jaundice. He was evaluated at multiple hospitals and initially diagnosed with liver abscess, prompting a computed tomography (CT) scan of his abdomen that revealed signs of chronic liver disease and multifocal lesions in both lobes of the liver suspicious for cholangiocarcinoma or hepatocellular carcinoma. Liver biopsy revealed poorly differentiated adenocarcinoma. Immunohistochemical workup showed tumor cells positive for CK7 and CK19, supporting a diagnosis of intrahepatic cholangiocarcinoma. Given the imaging findings of multifocal, liver-limited disease, the tumor was classified as AJCC clinical stage II intrahepatic cholangiocarcinoma. He was initially treated with first line systemic therapy consisting of gemcitabine 1000 mg/m^2^ and cisplatin 25 mg/m^2^ on days 1 and 8 of 21-day cycles, and durvalumab 1500 mg every three weeks for approximately four months. Declining liver function and progressive disease prompted a repeat biopsy to identify additional actionable molecular targets. Detection of HER2-low expression led to the addition of HER2-targeted therapy (trastuzumab deruxtecan (T-DXd), *Enhertu*, 6.4 mg/kg every 3 weeks). Four days after the first T-Dxd treatment, the patient presented for histotripsy with an ECOG performance status of 1 and elevated liver enzymes(alkaline phosphatase 465 U/L, ALT 104 U/L, AST 200 U/L, and bilirubin 5.9 mg/dL). Palliative histotripsy was performed under general anesthesia using real-time ultrasound guidance on one tumor in the left anterior lateral segment of the liver (segment 3, **Figure 1A**, red arrow). Treatment was performed at an average voltage of 26.8% to a planning treatment volume (PTV) of 13.0 cm^3^ with 0.5 cm margins, and full coverage of the targeted lesion was verified by post-procedure CT. The patient tolerated the procedure well without any peri-procedural complications and was discharged the same day. One-month post-procedure, magnetic resonance (MR) imaging demonstrated a significant reduction in the size of the targeted tumor (**Figure 1E**, green arrow). Distant liver tumors that were not treated by histotripsy (**Figures 1B–D**, green measurements) also exhibited a reduction in size at the one-month MRI (**Figures 1F–H**, yellow arrows), consistent with the effects of systemic treatment the patient was able to continue. Liver function also improved, with decreasing alkaline phosphatase 251 U/L and AST 43 U/L and normalizing bilirubin 1.0 mg/dL and ALT 28 U/L, allowing targeted drug therapy to continue.

**Figure 1:**
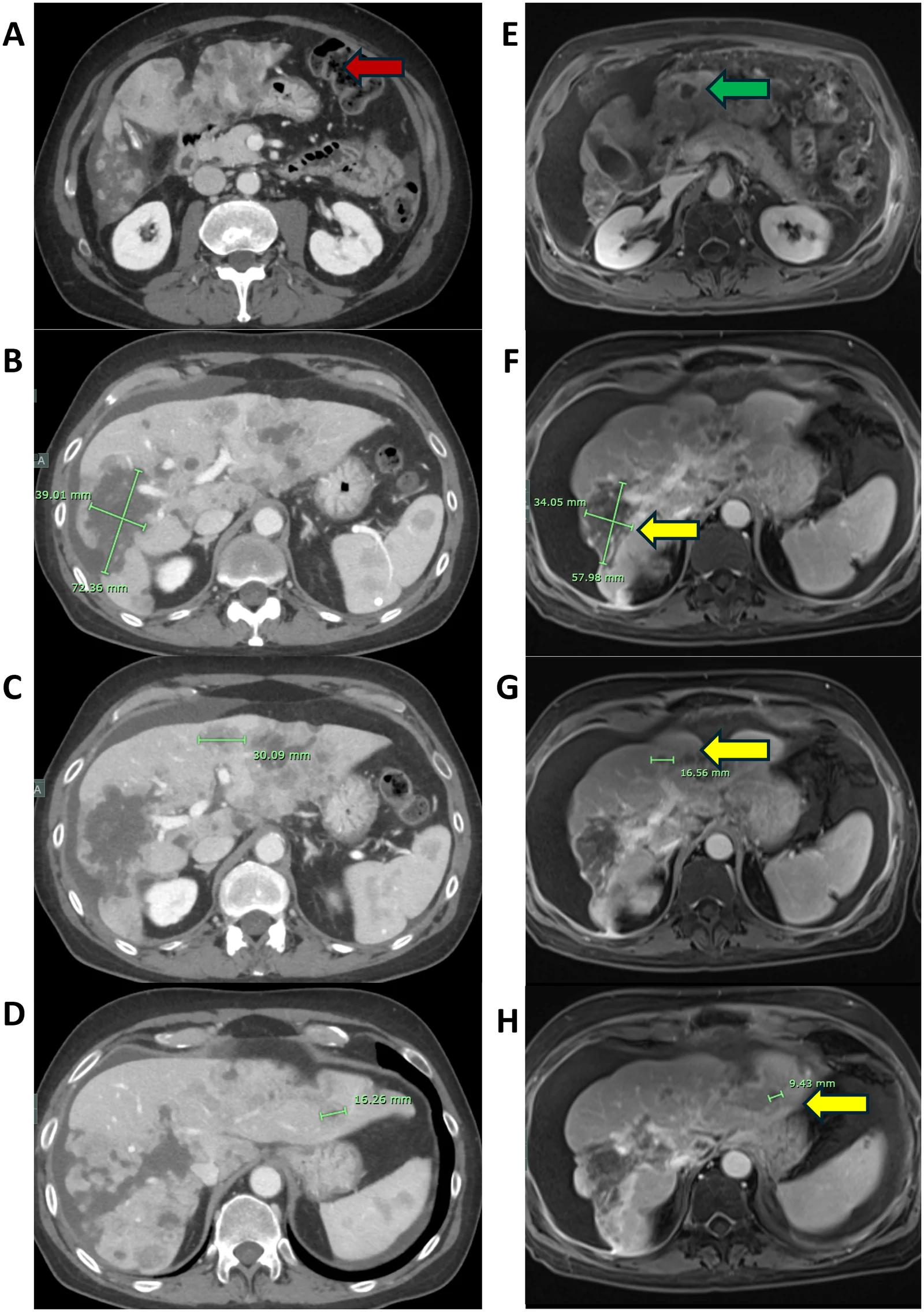
**(A)** Axial T1-weighted MR image of the liver before histotripsy treatment displaying the target tumor lesions in segment 3 (red arrow). **(B-D)** Extensive disease burden not planned for histotripsy. **(E)** Axial T1-weighted MR image one month post-procedure, showing marked reduction in tumor burden within segment 3 (green arrow). **(F–H)** One-month post-treatment MRIs showing notable reduction in non-treated tumors (yellow arrows).

A second histotripsy procedure was performed with the goal of completely treating five more lesions out of the remaining tumors (**Figure 2A**, red arrows). The segment 2, 5, and 6 lesions were treated at average voltages of 51.6, 43.4, and 51.5%, to PTVs of 33.5, 11.2, and 4.2 cm^3^, respectively, and the segment 7/8 and 8 lesions were treated at an average voltage of 47.3% to a PTV of 16.9 cm^3^. All treatments were performed with 5 mm margins. A paracentesis was concurrently performed to remove 2.5 L of serous ascitic fluid. The patient again tolerated the procedures well. Approximately 2 weeks later, liver function continued to improve with decreasing alkaline phosphatase 234 U/L and normalizing AST 42 U/L, bilirubin 13 umol/L, and ALT 37 U/L. CT imaging obtained the same day post histotripsy confirmed full coverage of the targeted lesion and displayed expected inflammatory response in the treated lesions (**Figures 2B, C**). Further imaging performed three months post procedure showed decreased enhancement and involution of the histotripsy treated area (**Figures 2D, E**, green arrows). Complete tumor response was demonstrated on positron emission tomography-computed tomography (PET/CT) imaging (complete absence of fluoro-deoxyglucose (FDG) uptake) performed at 4-month post histotripsy when compared with PET/CT images obtained at baseline (**Figure 3**). Given that this favorable response indicated sustained remission and the patient’s ECOG performance status had improved to 0, the patient was re-evaluated for a liver transplant. He stopped systemic therapy and underwent a liver transplant from a donor after circulatory death. Pathologic evaluation of the explanted liver revealed four tumor nodules measuring 3.7, 4.1, 4.6, and 7.8 cm in greatest dimension. All tumor nodules were entirely submitted for histopathologic evaluation. Sections demonstrated a pathologic complete response, characterized by absence of residual viable tumor in all examined sections, corresponding to AJCC/CAP stage ypT0N0. The tumor beds showed diffuse fibrosis, mild inflammation, and mild ductular reaction (**Figures 4A–C**). A timeline of the patient’s treatment course is shown in **Figure 4D**.

**Figure 2:**
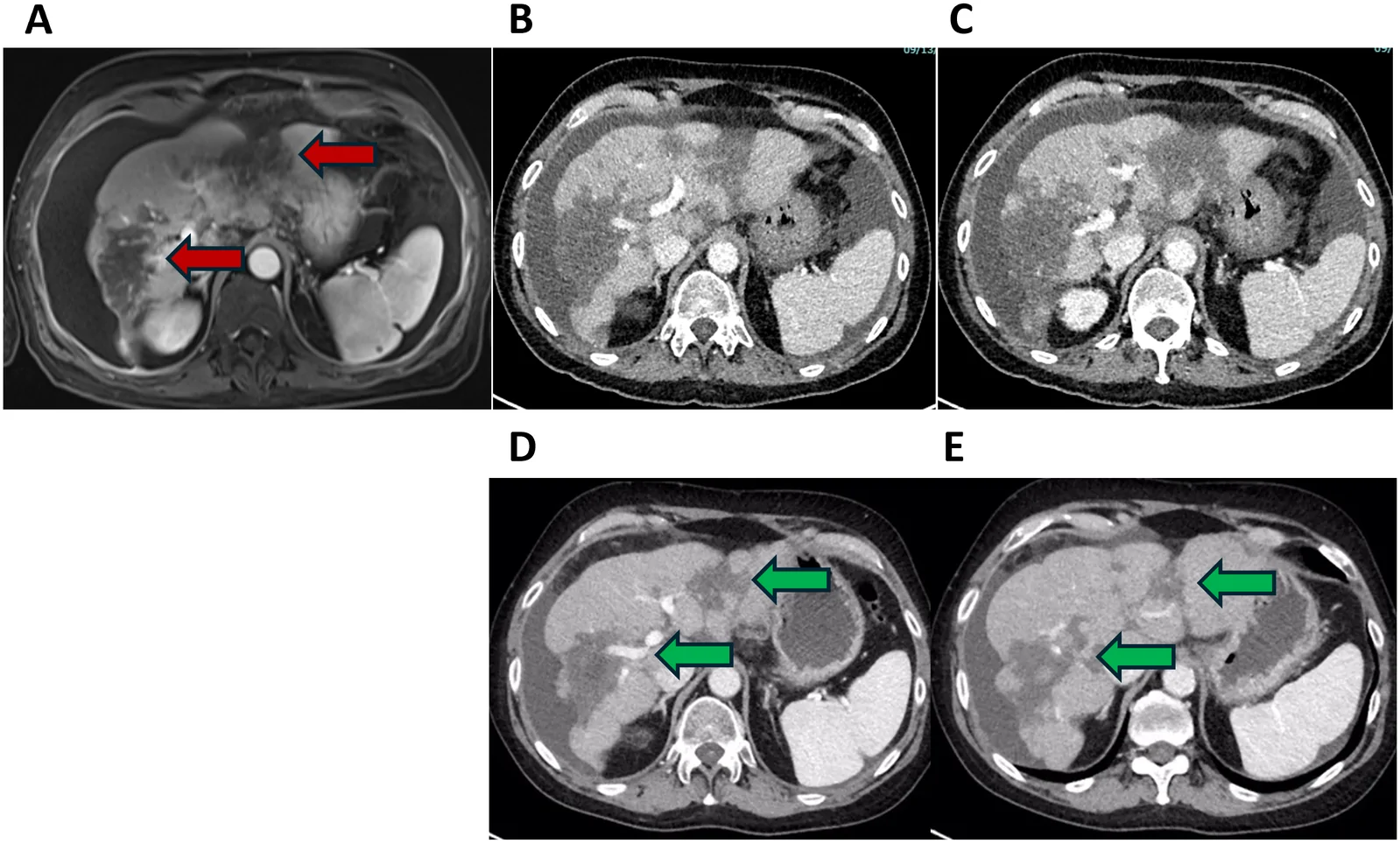
**(A)** Axial T1-weighted MR image of liver before a second histotripsy treatment displaying target tumor lesions in segments 2, 5, 6, 7/8, and 8 (red arrow). **(B, C)** Axial CT scans acquired within the same day after the second histotripsy procedure revealing expected inflammatory response in the treated lesions (green arrows). **(D, E)** Axial CT scans acquired 3 months after the second histotripsy procedure illustrating decreased enhancement and involution of the treated tumors.

**Figure 3:**
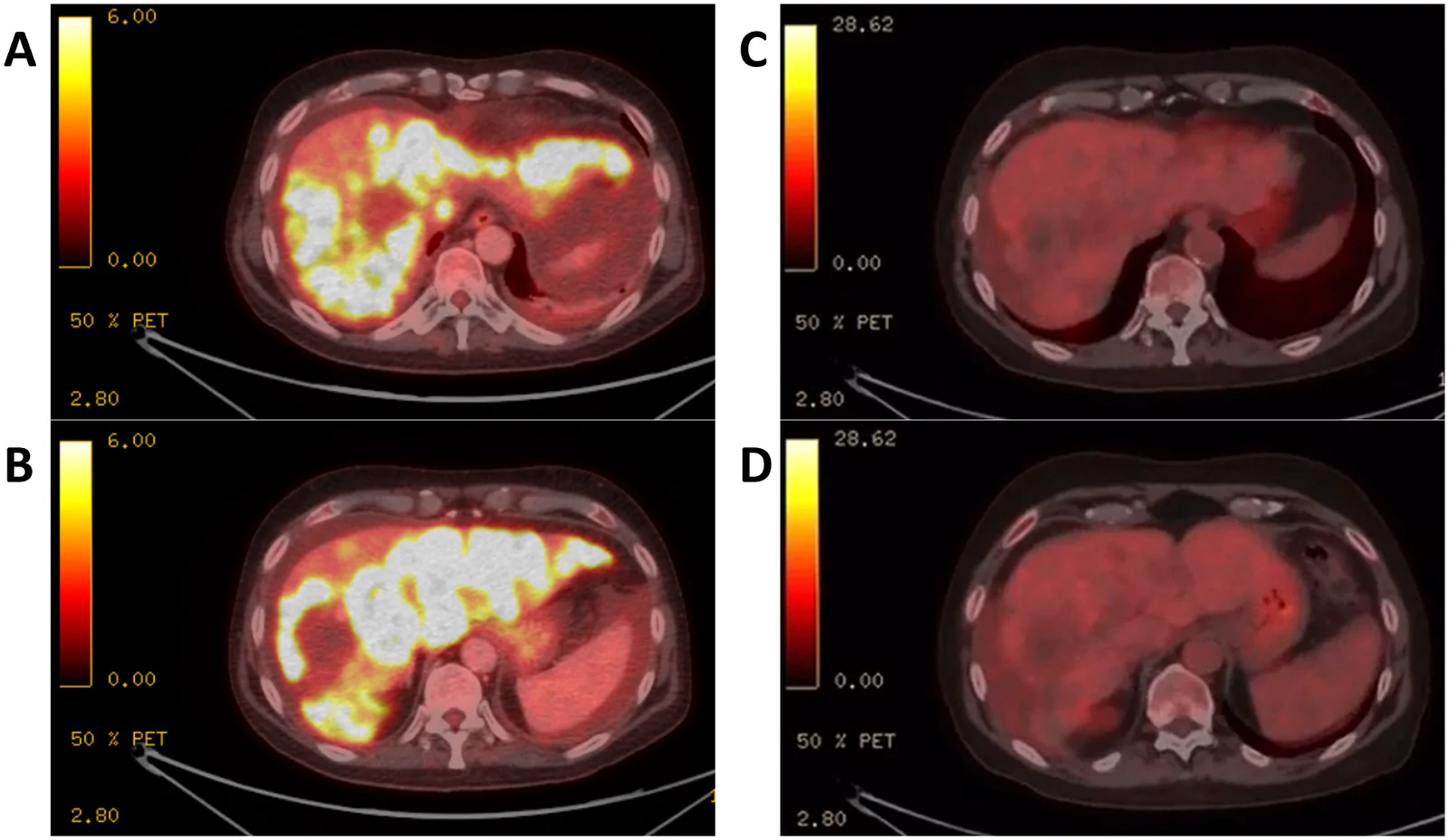
**(A, B)** Representative PET-CT images of the liver prior to histotripsy. **(C, D)** PET-CT scans acquired 4 months after the second histotripsy procedure.

**Figure 4:**
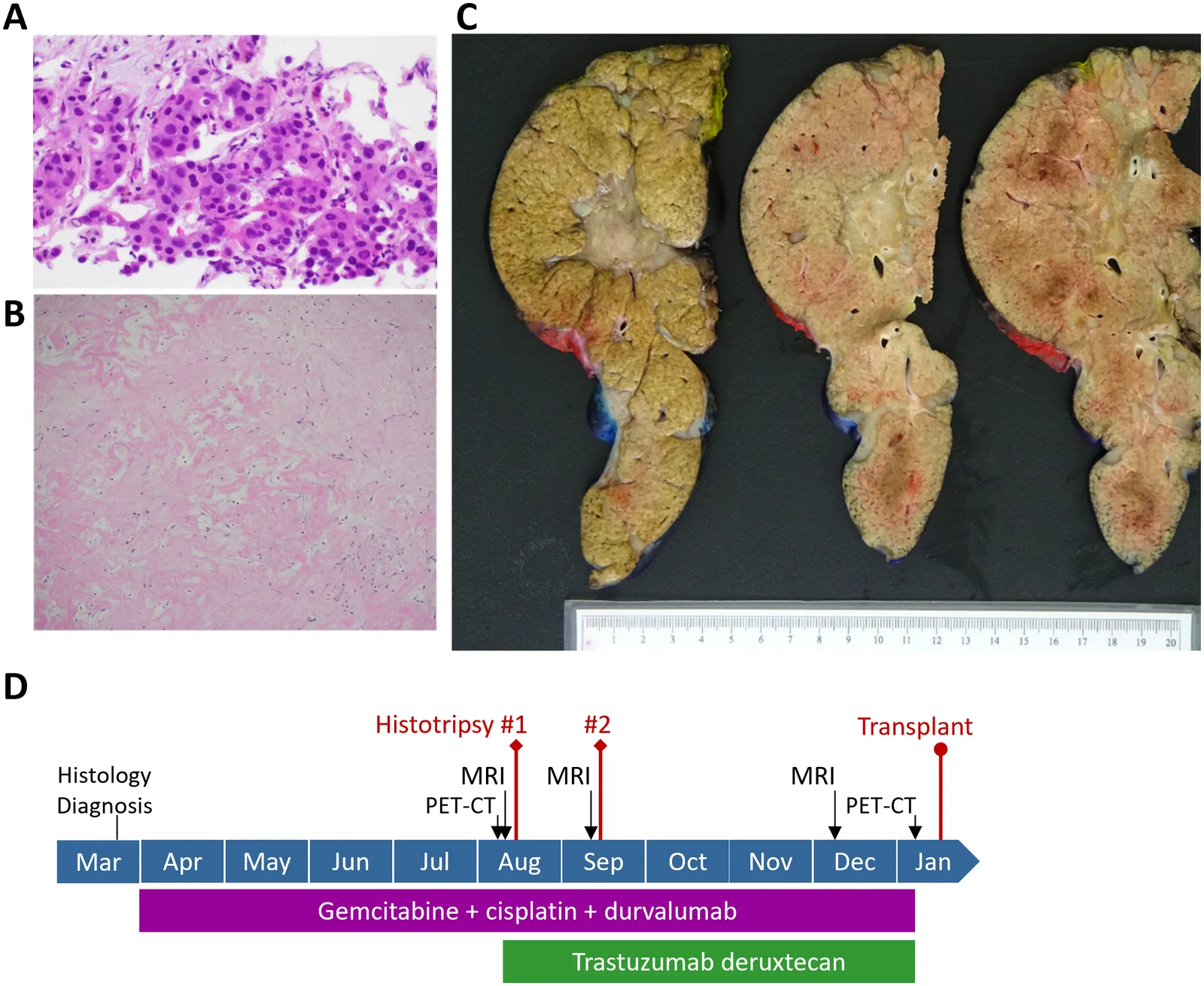
**(A)** Needle biopsy of intrahepatic cholangiocarcinoma prior to treatment (H&E, x200). **(B)** Histologic section from an explant tumor showing fibrosis and myxoid stroma with no viable tumor identified, consistent with a complete pathologic response (H&E, x100). **(C)** Liver explant demonstrating tumors with treatment effect characterized by diffuse myxoid change. **(D)** A schematic time course of the entire case.

## Patient perspective

The patient was asked about his expectations before the histotripsy procedure, how the results measured up, how the non-invasive treatment compared to previous procedures, and what motivated his decision to pursue histotripsy. “I went into the histotripsy procedure with almost no hope left- the only thing that provided even a glimmer of hope was this treatment. Just one week after the procedure I began to see improvement in everything (symptoms, labs, etc.), and day by day those improvements continued all the way up to the transplant. This noninvasive approach was unlike any of the traditional procedures I’d experienced before, there truly was no comparison. I didn’t notice any side effects at all with histotripsy. My family and I chose this option because we had run out of alternatives, and ultimately, it turned out to be the best treatment I could have received.”

## Discussion

Intrahepatic cholangiocarcinoma remains an aggressive malignancy with limited curative options and a high risk of intrahepatic progression, underscoring the need for strategies that achieve durable locoregional control, particularly in patients who might otherwise be ineligible for resection or transplantation. Here, we report on a patient who underwent successful liver transplant and exhibited complete necrosis of the tumors at explant pathology following systemic treatment with chemotherapy, HER2-targeted therapy, and histotripsy. This case is similar to that of another patient, this time with hepatocellular carcinoma (11), who also underwent a successful liver transplantation, thereby highlighting the potency of histotripsy, alone or in combination with systemic therapy, and its potential use as a bridging therapy for patients who may be initially ineligible for transplant.

Beyond histotripsy-induced local tumor control, the apparent responses of distant liver lesions that were not treated by histotripsy in this patient raises the possibility of an immunomodulatory contribution from histotripsy. Evidence of an “off-target” effect attributed to histotripsy has so far been reported anecdotally in humans (12, 13). Yet, preclinical studies have described the ability of histotripsy to trigger the release of tumor-associated antigen in a dose-dependent manner, promote immunogenic cell death, induce inflammatory and antigen-presenting cell populations within ablated tumors, and increase the number of circulating tumor-specific CD8+ T cells (14–17). It is therefore possible that, as demonstrated in pre-clinical studies, this patient benefited from the synergy between histotripsy and immunotherapy resulting in a highly significant immune-mediated response. Dedicated clinical studies are needed to confirm such results.

This case report has limitations inherent in the fact that it is by definition a single case. As a result, it is difficult to draw meaningful conclusions. However, the end-result of complete pathologic response at liver explant following a well-tolerated local therapy supports further evaluation of histotripsy, alone or in combination with systemic therapy as a potential therapy for patients with advanced cholangiocarcinoma. Such findings are encouraging and certainly warrant further investigations into specific conditions that may enhance the immunomodulatory potential of histotripsy.

## Conclusion

This case highlights the potential utility of histotripsy as an effective bridging therapy for patients with intrahepatic cholangiocarcinoma to successfully undergo liver transplantation.

## Data Availability

The raw data supporting the conclusions of this article will be made available by the authors, without undue reservation.

## References

[1] Brindley PJ Bachini M Ilyas SI Khan SA Loukas A Sirica AE . Cholangiocarcinoma. Nat Rev Dis Primers. (2021) 7:65. doi: 10.1038/s41572-021-00300-2 34504109 PMC9246479

[2] Oh DY He AR Bouattour M Okusaka T Qin S Chen L . Durvalumab or placebo plus gemcitabine and cisplatin in participants with advanced biliary tract cancer (TOPAZ-1): updated overall survival from a randomised phase 3 study. Lancet Gastroenterol Hepatol. (2024) 9:694–704. doi: 10.1016/S2468-1253(24)00095-5 38823398

[3] Kelley RK Ueno M Yoo C Finn RS Furuse J Ren Z . Pembrolizumab in combination with gemcitabine and cisplatin compared with gemcitabine and cisplatin alone for patients with advanced biliary tract cancer (KEYNOTE-966): a randomised, double-blind, placebo-controlled, phase 3 trial. Lancet. (2023) 401:1853–65. doi: 10.1016/S0140-6736(23)00727-4 37075781

[4] Xu Z Hall TL Vlaisavljevich E Lee FT Jr . Histotripsy: the first noninvasive, non-ionizing, non-thermal ablation technique based on ultrasound. Int J Hyperthermia. (2021) 38:561–75. doi: 10.1080/02656736.2021.1905189 33827375 PMC9404673

[5] Xu Z Khokhlova TD Cho CS Khokhlova VA . Histotripsy: a method for mechanical tissue ablation with ultrasound. Annu Rev BioMed Eng. (2024) 26:141–67. doi: 10.1146/annurev-bioeng-073123-022334 38346277 PMC11837764

[6] Uysal M Wehrle CJ Satish S Knott E Hong H Allkushi E . Histotripsy of liver tumors: patient selection, ethical discussions, and how we do it. Cancers. (2025) 17:1100. doi: 10.3390/cancers17071100 PMC11987918 40227626

[7] Vidal-Jove J Serres X Vlaisavljevich E Cannata J Duryea A Miller R . First-in-man histotripsy of hepatic tumors: the THERESA trial, a feasibility study. Int J Hyperthermia. (2022) 39:1115–23. doi: 10.1080/02656736.2022.2112309 36002243

[8] Mendiratta-Lala M Wiggermann P Pech M Serres-Creixams X White SB Davis C . The #HOPE4LIVER single-arm pivotal trial for histotripsy of primary and metastatic liver tumors. Radiology. (2024) 312:e233051. doi: 10.1148/radiol.233051 39225612 PMC11427859

[9] Wehrle CJ Burns K Ong E Couillard A Parikh ND Caoili E . The first international experience with histotripsy: a safety analysis of 230 cases. J Gastrointest Surg. (2025) 29:102000. doi: 10.1016/j.gassur.2025.102000 39978577

[10] Ziemlewicz TJ Critchfield JJ Mendiratta-Lala M Wiggermann P Pech M Serres-Creixmas X . The #HOPE4LIVER single-arm pivotal trial for histotripsy of primary and metastatic liver tumors: 1-year update of clinical outcomes. Ann Surg. (2025) 282(6):908–16. doi: 10.1097/SLA.0000000000006720 40201962 PMC12594125

[11] Uysal M Wehrle CJ Coppa C Kamath S Krishnamurthi S Martin C . Bridging therapy with histotripsy prior to liver transplantation for hepatocellular carcinoma: a first case report. Exp Hematol Oncol. (2025) 14:20. doi: 10.1186/s40164-025-00604-z 40001149 PMC11863397

[12] Vidal-Jove J Serres-Creixams X Ziemlewicz TJ Cannata JM . Liver histotripsy mediated abscopal effect-case report. IEEE Trans Ultrason Ferroelectr Freq Control. (2021) 68:3001–5. doi: 10.1109/tuffc.2021.3100267 34310299

[13] Pepple AL Guy JL McGinnis R Felsted AE Song B Hubbard R . Spatiotemporal local and abscopal cell death and immune responses to histotripsy focused ultrasound tumor ablation. Front Immunol. (2023) 14:1012799. doi: 10.3389/fimmu.2023.1012799 36756111 PMC9900174

[14] Worlikar T Zhang M Ganguly A Hall TL Shi J Zhao L . Impact of histotripsy on development of intrahepatic metastases in a rodent liver tumor model. Cancers (Basel). (2022) 14(7):1612. doi: 10.3390/cancers14071612 35406383 PMC8996987

[15] Qu S Worlikar T Felsted AE Ganguly A Beems MV Hubbard R . Non-thermal histotripsy tumor ablation promotes abscopal immune responses that enhance cancer immunotherapy. J Immunother Cancer. (2020) 8(1):e000200. doi: 10.1136/jitc-2019-000200 31940590 PMC7057529

[16] Hay AN Vickers ER Patwardhan M Gannon J Ruger L Allen IC . Investigating cell death responses associated with histotripsy ablation of canine osteosarcoma. Int J Hyperthermia. (2023) 40:2279027. doi: 10.1080/02656736.2023.2279027 38151477 PMC10764077

[17] Tang S McGinnis R Cao Z Baker JR Jr Xu Z Wang S . Ultrasound-guided histotripsy triggers the release of tumor-associated antigens from breast cancers. Cancers (Basel). (2025) 17(2):183. doi: 10.3390/cancers17020183 39857965 PMC11764245

